# Towards carbon neutrality: mapping mass retrofit opportunities in Cambridge, UK

**DOI:** 10.1098/rsos.241337

**Published:** 2025-01-15

**Authors:** Humberto Mora, Ronita Bardhan

**Affiliations:** ^1^ Cambridge Institute for Sustainability Leadership (CISL), University of Cambridge, Cambridge CB2 1TN, UK; ^2^ Sustainable Design Group, Department of Architecture, University of Cambridge, Cambridge CB2 1PX, UK

**Keywords:** hard to decarbonize, multi-variate clustering, thermal imaging, archetypes, mass retrofit

## Abstract

This study proposes a methodology and a proof of concept to target and prioritize mass retrofitting of residential buildings in the UK using open building datasets that combine fabric energy efficiency and fuel poverty to meet the net-zero targets. The methodological framework uses a series of multi-variate statistical and geospatial methods that consider urban, socio-economic and physical attributes. In addition, thermal imaging is implemented to provide insights at the building scale. We define a hard-to-decarbonize (HtD) metric to enable the clustering of different residential types to establish retrofitting priorities. Using Cambridge, UK, as a case study, five neighbourhoods were identified and characterized to help determine decarbonization intervention priorities. We found that one of five clusters of neighbourhoods is HtD and requires more policy support from government for the implementation of retrofit strategies. The achieved framework has the potential to inform policy and decision making. Of relevance, it is applicable to different urban contexts.

## 1. Introduction

In order to decarbonize the property sector, the UK Government signed a legally binding agreement to achieve net-zero (NZ) carbon by 2050. NZ can be defined as a reduction in demand for energy and materials to a level that can be sourced from systems that do not emit greenhouse gases [1]. To achieve the targets, the decarbonization of the domestic building stock is essential [2]. Strikingly, over 29 million homes in the UK must be made energy efficient by 2050 in line with these goals [3]. Therefore, *mass retrofit*, which considers the retrofit of multiple building units at the same time at a neighbourhood scale, has a crucial role to play in the reduction of the building stock carbon emissions [4].

The UK’s ageing residential building stock needs to be decarbonized en masse to achieve NZ targets. In England and Wales, over 20% of dwellings were built before 1929. In addition, most of the housing building stock in London and the East of England were built before the 1980s, where Cambridge is located [5]. Of note, hard-to-decarbonize (HtD) homes account for 25% of the existing residential stock carbon emissions in the UK [6]. To effectively define mass retrofit strategies, identifying and characterizing building typologies, including HtD, is fundamental to tailor retrofit at scale measures in these contexts.

Targeting urban areas and dwellings for retrofit at scale remains one of the biggest challenges in the decarbonization of the building stock [7]. The scale of retrofit priorities is context-specific and strategies to determine what is hard or easier to decarbonize are needed. Depending on the context and building category, multiple variables can be mapped including building period, energy performance certificates (EPCs), fuel poverty, socio-economic conditions, among others. Their inter-relationships can vary between different city areas due to intrinsic building fabric and population characteristics. Hence, this article investigates *what housing building stock should be targeted for mass retrofit in Cambridge*. In detail, the research evaluates what neighbourhoods and building typologies should be prioritized for mass retrofit and what potential policy strategies could be implemented.

Our work contributes to both the academic and the urban leadership fields. The framework presented here aims to target the profiles of homes that can be grouped together for different mass retrofit opportunities. Through a multi-variate approach, it introduces the holistic integration of socio-economic and built environment physical attributes. Simultaneously, it focuses on both neighbourhood- and building-scale data. Importantly, we define an HtD metric to enable the clustering of different residential types to establish retrofit priorities. The resulting framework can inform policy and decision making by characterizing neighbourhood and building attributes. Ultimately, the outcome interpretation has the potential to guide city leaders and retrofit intervention policy through various decarbonization pathways [3,4,8–11].

### 1.1. Background literature

*Mass retrofit* or *retrofit at scale*, defined as the retrofit of multiple dwellings at community and city scale [7], is increasingly identified as a much-needed step towards achieving NZ targets [12]. Yet, it has not been achieved at an accurate volume and standard [13]. Some argue that this is due to flaws in policy and governance [4], lack of skills or limitations in the supply chain [14].

The urgent need for the upscale of retrofitting can be justified, to some extent, by global and UK projections. The International Energy Agency (IEA) developed the Efficient World Scenario (EWS) which projects a target of a 40% increase in energy efficiency compared with current levels. It assumes the upgrade of the built stock with more efficient mechanical systems and building fabric improvements [15,16]. Similarly, projections in the UK assume the acceleration of fabric improvements and low-carbon technologies in the 2030s, the intervention of remaining hard-to-treat homes by the 2040s and the decarbonization of the domestic building stock to meet NZ targets by 2050 [2].

Current retrofit rates are too slow to achieve NZ decarbonization targets and, hence, the urgency to legislate appropriate policies [17]. Strikingly, the scale of the challenge represents about 1.6 homes needing retrofit per minute [3,7]. Not surprisingly, multiple reviews of the subject align in stating that the pace required to meet the goals is not being followed [3,4,18] which highlights an imminent need to accelerate retrofit.

The entire neighbourhood scale is fundamental to achieve a larger, better-performing built stock. Indeed, it has been demonstrated that building-only interventions are not sufficient to achieve holistic impacts in urban areas. Therefore, whole neighbourhood energy systems and community dynamics should be targeted to achieve economy of scale and to capitalize on energy systems [19].

### 1.2. The UK building stock and hard-to-decarbonize homes

The UK residential sector’s single-family housing accounts for 80% of the building stock, with only 20% multi-family buildings. This differs from other European countries where the share is evenly distributed between both residential types such as Austria or France [20]. Furthermore, historic buildings are part of the existing building stock that requires upgrades to meet NZ targets and present singularities in terms of possible retrofit levels of intervention. About 20–30% of the UK domestic stock have some form of heritage value [21–23] and some can be HtD. HtD refers to homes with intrinsic physical or occupant-related characteristics that limit deploying decarbonization measures [8]. Examples include properties with heritage status, off-grid houses, those with hard-to-reach (HtR) occupants or hard-to-treat (HtT) homes, among others (table 1). HtD buildings tend to have an EPC rating of F–G, with up to 60–80% incidence, and above average levels of fuel poverty, which is the ability to afford to heat and cool homes to a comfortable level [24]. A recent study by Sun & Bardhan [25] has demonstrated that low intensity contextual data can be effective in identifying HtD buildings.

**Table 1.** HtD limiting attributes as currently defined (adapted from [8]).

HtD building typology	limiting attributes for retrofit
HtT homes	uninsulated solid non-standard cavity walls limit fabric intervention
HtR occupants	difficult to engage occupants with decarbonization plans
heritage status	limited options to fabric intervention
off-grid homes	difficult to access and include in urban retrofit strategy
homes with space constraints	modern systems like air source heat pumps cannot be installed
multi-occupancy high-rise blocks	hard and costly to insulate/possible fire risks

Apart from the HtD characteristics, there are multi-variate contextual factors that make homes vulnerable to decarbonization. These may include the geospatial location of these homes which impact the local micro-climates and add temperature stresses [26,27], contextual and demographic factors like multiple indices of deprivation. The combination of these factors organically generates archetypes of HtD homes that will require distinct interventions to ease the path to decarbonization. Understanding these archetypes is crucial to inform and shape mass retrofit strategies and whether they can meet broader performance targets in line with NZ goals. The subsequent section illustrates the methodology adapted for identifying and generating the archetypes of HtD homes.

### 1.3. Multi-variate approach

The integration of variables is fundamental to achieve a holistic overview of an urban context. Previous studies demonstrated that this can be done through the implementation of a sequence of statistical methods. Bardhan *et al.* [28] assessed the relationship between a compact city and quality of life through a multi-variate approach. Specifically, they implemented quantitative methods that enable the understanding of the relationship between individual data components, informing possible ways to group urban areas and their associated characteristics. That study was one of the first to reliably apply the cluster enrichment approach, a method typically used to characterize lists of genes in the biological field, in urban studies to develop archetypes. Our work builds on the methodology developed in that precedent study and pivots the combination of variables to inform mass retrofitting.

## 2. Methodology and data

Mass retrofit entails multiple aspects of the built environment including building fabric, environmental and socio-economic performance, among others. These can be assessed holistically at different scales and multiple variable combinations can be considered. As a result, based on the inputs analysed, a range of outcomes can be expected to inform different aspects of retrofit at scale.

**Figure 1.**
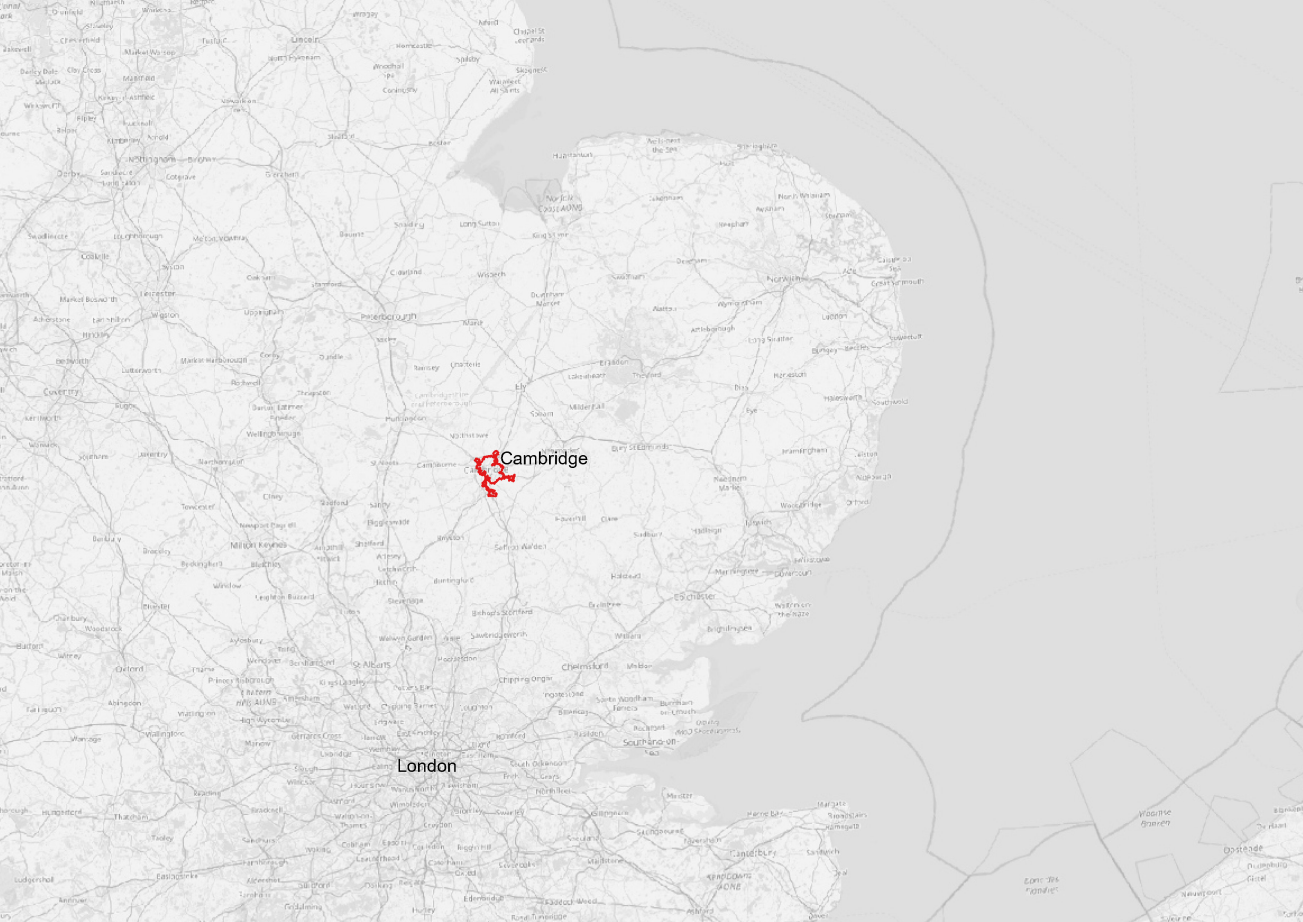
Cambridge, UK, location.

Cambridge, UK, is used as a case study (figure 1). Here, the residential decarbonization challenge is, to an extent, related to the aged existing residential stock. With a predominant single-housing typology, most homes were built before the 1980s, and it is estimated that about one-third of homes are dated before the 1930s [29]. Of interest, a recent study that employed a multi-source data approach to identify HtD in the same city used EPC data and estimated that the most common criteria in the HtD stock were solid walls, followed by flat roofs and heritage value [25].

### 2.1. Targeting neighbourhoods for mass retrofitting

#### 2.1.1. Data

**Figure 2.**
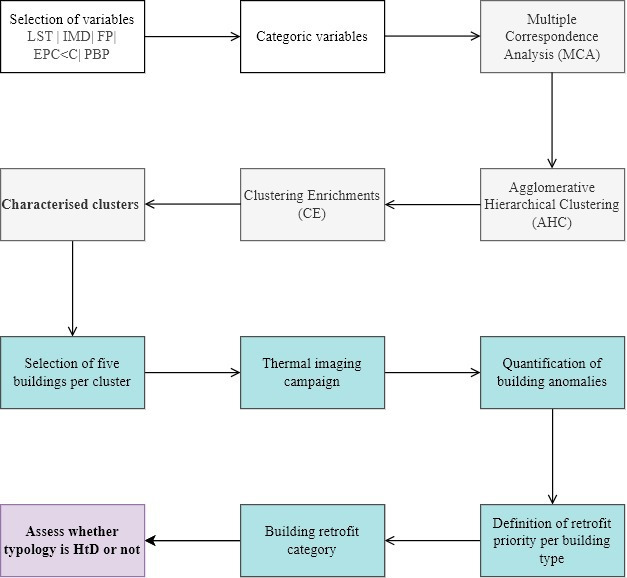
Methodological framework. Grey boxes indicate methods and steps considered at the neighbourhood scale. Blue boxes indicate methods and steps considered at the building scale. Purple box refers to the final step where both neighbourhood and building scale data are integrated to determine whether a building typology is HtD.

A multi-variate dataset was used to characterize the houses for mass retrofit needs and to inform possible clusterings for interventions (figure 2). Specifically, it allowed assessment of the combined influence of both physical and socio-economic attributes. The unit base for data collection was layer super output areas (LSOAs) which are geographical, also referred to as neighbourhoods [30] (electronic supplementary material, figure S1). A holistic set of five open-source variables was defined for Cambridge (table 2; and electronic supplementary material, figure S2).

**Table 2.** Sources for each of the variables considered in the analysis.

variable	source
land surface temperature	Landsat 8 in Google Earth Engine
index of multiple deprivation (IMD)	public IMD from 2019
percentage of fuel poor households	BEIS 2022 Fuel Poverty Database
percentage of homes with EPC below C (EPC < C)	public ONS data from 2021 census
predominant building period (PBP)	dwelling ages and prices from Consumer Data Research Centre (CDRC)

#### 2.1.2. Land surface temperature

Land surface temperature (LST) is defined as a metric of the Earth’s temperature at touch [31]. It has become increasingly relevant in the study of urban micro-climate mapping, allowing to identify which city areas absorb more heat than others [32]. Landsat 8 satellite imagery was used to determine LST of Cambridge in the warmest two weeks of the year 2022, when the historical highest hottest day was recorded, using the open-source application Google Earth Engine (GEE). It is considered as an indicator of heat stress throughout the city fabric.

#### 2.1.3. Index of multiple deprivation

Index of multiple deprivation (IMD) is a measure of relative deprivation in England, and it is based on an established framework that considers income, employment, health deprivation and disability, education, crime, barriers to housing and services and living environment [30]. IMD deciles (1 referring to the most deprived) were collected for Cambridge LSOAs.

#### 2.1.4. Percentage of households that are fuel poor

Percentage of households that are fuel poor (FP) is representative of whether occupants are able or not to heat and cool their homes at comfortable levels [24]. Typically, a household is considered to be fuel poor if the energy efficiency rating is below C and if its disposable income (income after housing costs and energy needs) would be below the poverty line. In detail, it considers a disposable income of less than 60% of the national median [33]. Developed by [33], a database that considered the distribution of fuel poor households by LSOA was used for this study based on 2020 data.

#### 2.1.5. Percentage of homes with energy performance certificates below C

Percentage of homes with EPCs below C (%EPC < C) is a metric of energy performance [34]. The purpose of the EPC is to determine how energy efficient a building is. It is calculated based on multiple factors including building typology, age of the building, dimension, glazing ratios, type of wall and glazing, among others. The rating is provided in a scale of G to A, where G is the least efficient and A is the most efficient [34]. Based on public data by the ONS, the ratio of homes with an EPC below C was used.

#### 2.1.6. Predominant building period

Predominant building period (PBP) refers to the period in which the building was built which is available in multiple year bands. Based on a count, the predominant building age range per LSOA was estimated. The age bands were divided into categories in line with those identified by the ONS in electronic supplementary material, figure S2. Based on public data from the CDRC, the predominant building stock (BS) periods were visualized in QGIS.

#### 2.1.7. Converting numeric to categoric variables

Following the collection of numerical attributes, the different variables described above were converted into categorical variables based on a 1–5 scale as shown in electronic supplementary material, table S1. In addition, the overall count of categories was made using ‘R’ to analyse the overall data distribution. Further, statistical analysis was conducted using the categorical data.

#### 2.1.8. Multiple correspondence analysis

To explore the inter-relationship between the different variables, a multiple correspondence analysis (MCA) was developed. MCA is a statistical method that helps identify patterns in large datasets which can be described by locating each unit of analysis or variable as a point in a two-dimensional space [35]. In addition, the MCA allowed the determination of the dimensions that provided the most relevant information, based on principal inertias (variation), individual LSOA contributions and quality of representation.

#### 2.1.9. Agglomerative hierarchical clustering

To group the different LSOAs based on their similarity, an agglomerative hierarchical clustering (AHC) was carried out. This approach considers the categorical results for the different variables associated with each LSOA. The algorithm uses the Euclidean distances between each pair of components to determine the cluster [36]. In this case, five clusters were defined.

#### 2.1.10. Cluster enrichment technique based on an over-representation analysis

To characterize the clusters, a cluster enrichment (CE) technique based on an over-representation analysis (ORA) was followed. CE is broadly used in biological sciences to define patterns in lists of genes. Specifically, it is used to determine whether a set of genes is statistically over-represented in a list [37]. The approach has been used in urban studies and was demonstrated in a novel study that looked at urban compactness and quality of life in a given city based on a multivariate approach [28].

The ORA is used to assess the statistical over-representation of a variable category in the given sample through tests like the *χ*^2^-test or hypergeometric distribution (HD). Here, it determines the probability of a group of variable categories occurring in one cluster and tests the distribution with a null hypothesis. The HD statistic is estimated based on the following equation:


$$p\left(x\right|N,n,mi)=\frac{\left(\begin{array}{c} m\\ x \end{array}\right)\left(\begin{array}{c} N-m\\ n-x \end{array}\right)}{\left(\begin{array}{c} N\\ x \end{array}\right)},$$

where *N* is the size of the whole sample, *m* is the size of the cluster *i*, *n* is the size of the functional group within the whole sample and *x* is the size of the functional group within the cluster *i*.

#### 2.1.11. Cluster enrichment interpretation

To inform ranges of priorities to implement retrofit policy, the CE analysis by variable follows a 1−5 scale, from very low to very high scale. Here, for all the variables, the higher the category, the more urgent the need for attention is from a retrofit perspective. Importantly, to tailor interventions, all priority levels could have a different interpretation from a leadership perspective.

#### 2.1.12. Paths to decarbonization

To inform retrofit urban policy, four general retrofit strategies are pre-defined to guide the CE interpretation. First, a fabric-first approach (FFA), which refers to the treatment of residential units envelope including glazing systems and solid wall areas, is relevant when energy efficiency or heat loss issues are encountered. It is typically argued that best practice shall follow a FFA before introducing carbon efficient technologies and that projects must be tailored to building typology and specific context conditions [38,39]. Second, technology interventions, which refer to the implementation of systems at scale such as roof treatment or heat pumps, may be pertinent in areas that present thermal stress. Third, financial subsidy shall be considered by local authorities in the most deprived areas following IMD and FP indicators to support the population to retrofit. Finally, making visible is relevant where other high priorities are not evidenced but awareness related to retrofit is still relevant. Overall, these strategies are used as a guide and could be further subdivided or redefined based on the specific application.

### 2.2. Building scale assessment

#### 2.2.1. Building data

Following MCA, AHC and CE, five building units were identified to conduct further building scale analysis for each cluster. Where possible, the selection was based on similar orientation, footprint and the residential typology (e.g. mid-terrace, semi-detached, detached, end-terrace). To inform the housing units, public information on the ‘Energy Performance of Building Data: England and Wales’ site by the Department for Levelling Up was taken into consideration where available. This included attributes like age, fabric construction type and typology. Of note, for a building to be in this database, it needs to have an EPC. Importantly, to preserve the privacy of residents, the houses’ addresses were anonymized. The fabric performance was assessed using an infrared (IR) thermal imaging approach.

#### 2.2.2. Thermal imaging

IR is a non-intrusive technique employed to analyse the thermal performance of structural or non-structural building fabric components. It allows for immediate access to raw surface temperature and for the identification of areas of thermal bridges, the quality of thermal insulation or moisture patterns [40,41]. The identification of defects through this technique is based on the surface thermal difference.

#### 2.2.3. Defining a house need for retrofit

**Figure 3.**
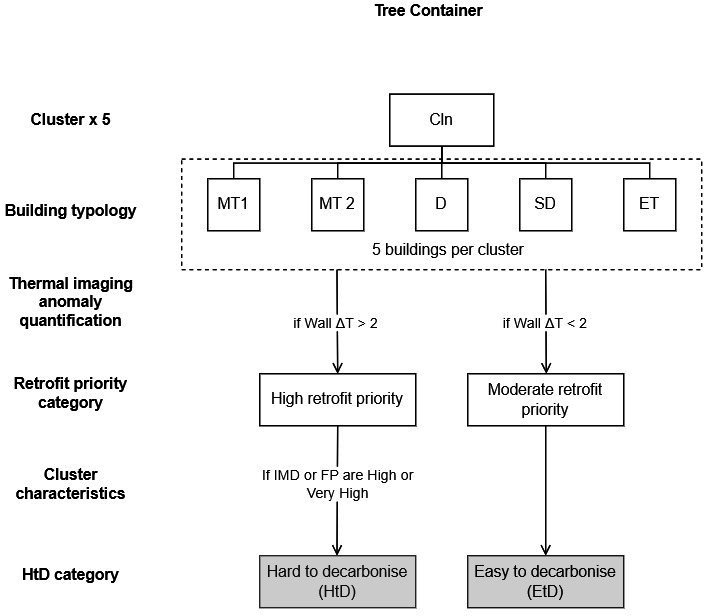
Integration of data and steps to determine whether a given building typology in a cluster is HtD or not. MT: mid-terrace house; D: detached house; SD: semi-detached house; ET: end-terrace house.

Based on its potential to inform housing retrofit needs, the wall temperature difference (Δ*T*) was used as the main criterion to establish building-type retrofit priorities. Through the post-processing of thermal images in the software FLIR tools, Δ*T* was defined for the different components including glass, interfaces and solid wall (electronic supplementary material, table S3). Specifically, where wall Δ*T* > 2°C, the need for retrofit was classified as ‘High’ (figure 3).

### 2.3. Defining hard to decarbonize through the integration of multi-scale data

The overlay of data analysis at the neighbourhood and building scale was used to determine whether a building typology is HtD or not (figure 3). This was done by considering key aspects of the LSOA cluster and building type characterization. Depending on whether a building was found to have a high retrofit need and was located in a cluster where IMD or FP were high or very high, it was classified as HtD or not.

#### 2.3.1. Assessing trends

To contextualize building data observation, the dwelling analysis was assessed against commonly used metrics. First, for the same typology in each cluster, the facade Δ*T* was compared. Second, the same measurement was compared in terms of EPC rating and building age. Overall, this helped identify patterns in the sample studied.

## 3. Results

To determine what building stock should be targeted for mass retrofit, a data-driven approach was followed at both the neighbourhood and the building scale. First, a holistic multi-variate analysis was undertaken. Specifically, a set of variables was defined including LST, IMD, percentage of households in FP, EPC < C and the PBP. Each of these was categorized on a five-level scale relative to Cambridge, from very low to very high, where the highest indicates a need for attention. The variable categories were visualized using LSOAs as the geographical unit and a series of statistical methods was implemented to identify priority groups. Second, 25 buildings of different typologies were selected to conduct a thermal imaging analysis with a focus on the living room areas. The variation of temperature in different parts of the envelope was quantified, including the glazed area, the interfaces and the solid area. The latter was used to inform the level of urgency for retrofit. Finally, the overlay of data at both scales informs what building stock is HtD.

### 3.1. Five clusters defined in Cambridge

#### 3.1.1. Multiple correspondence analysis and agglomerative hierarchical clustering

**Figure 4.**
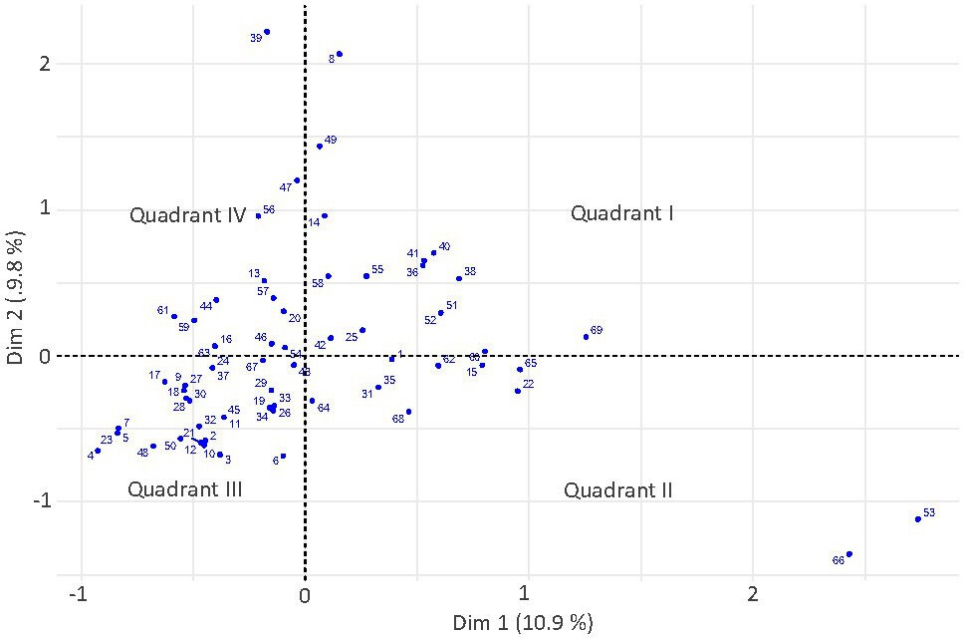
MCA LSOA plot shows each LSOA in a two-dimensional space. Numbers allocated to each LSOA correspond to the order by which they were retrieved from the ONS 2011 LSOA database.

**Figure 5.**
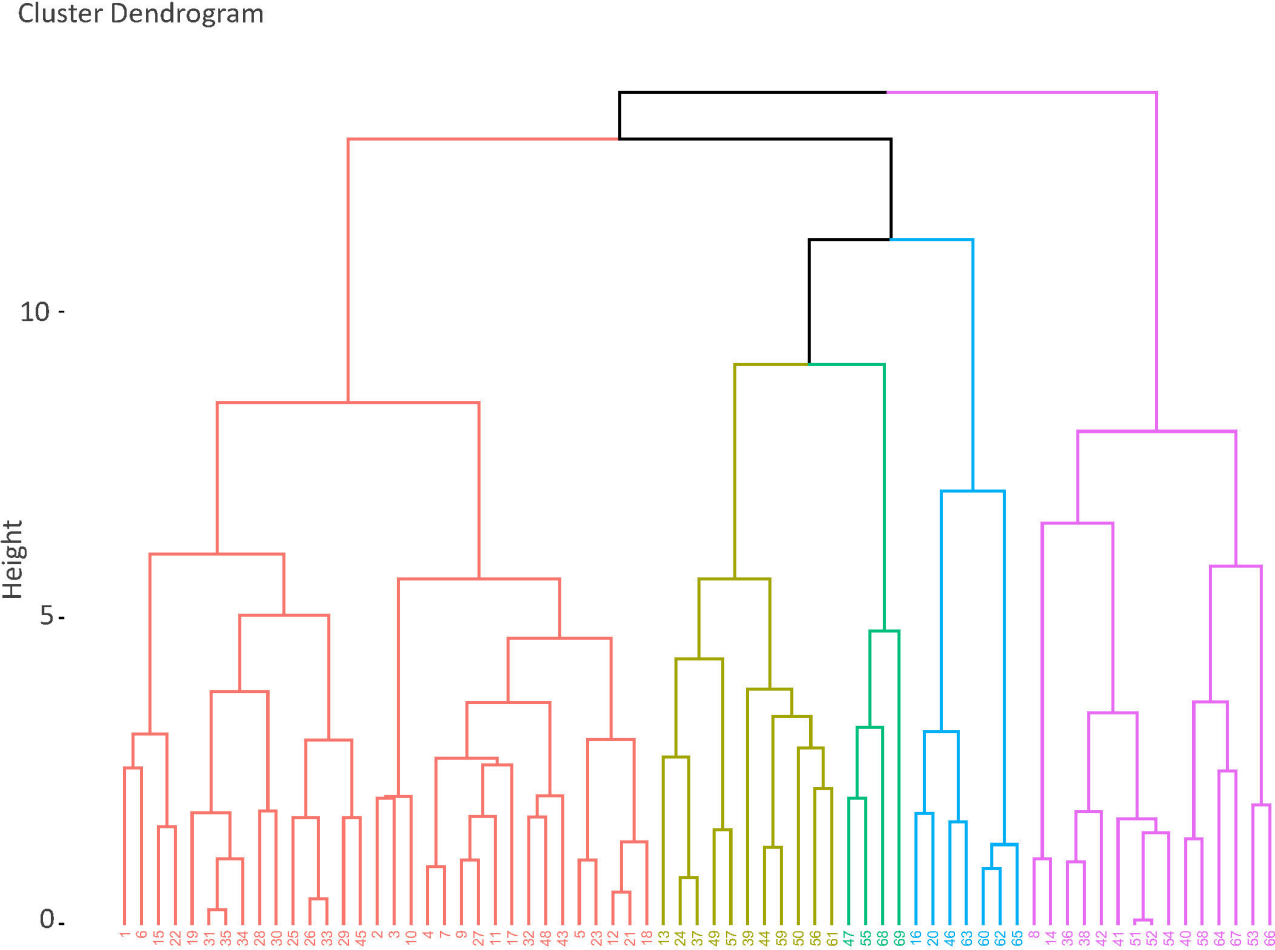
Cluster dendrogram based on AHC.

In order to visualize the relationship between all the Cambridge LSOAs based on five-variable categories, the dimensionality of the dataset was reduced through an MCA (figure 4). Furthermore, to group the different LSOAs, an AHC was carried out (figure 5). Five groups are defined in the context of Cambridge based on the statistical analysis and on contextual and spatial distribution. Cluster 1 is the largest in terms of both geographic area and number of LSOAs, followed by clusters 2, 3, 4 and 5.

#### 3.1.2. Cluster enrichment analysis

To characterize and help inform priority groups for mass retrofit, a CE analysis was conducted. Based on an over-representation analysis that used a hypergeometric distribution, statistically under-represented and over-represented variable categories were identified based on a two-tailed *p*-value as shown in electronic supplementary material, table S4. Where *p *< 0.05, the variable category is considered to be over-represented. Cluster 1 is characterized by an over-representation of LST medium, of IMD medium to very high, of FP low to very high, of EPC<C low to high and of PBP low to medium. Cluster 2 is defined by an over-representation of EPC<C and PBP very low and very high. Cluster 3 is characterized by an over-representation of LST very low and very high, IMD very low, FP low and EPC<C high. Cluster 4 shows an over-representation of IMD very low, FP very low, EPC<C low and high, and PBP low. Cluster 5 is characterized by an over-representation of LST high and very high, FP low, of EPC<C very low and PBP medium and high.

#### 3.1.3. Retrofit priorities at the building scale

A thermal imaging campaign was undertaken for five buildings in each cluster as shown in electronic supplementary material, figure S3. These were representative of different typologies including mid-terrace, semi-detached, detached and end-terrace. The focus of the analysis was on a portion of the building unit facade expected to correspond to the living room.

**Figure 6.**
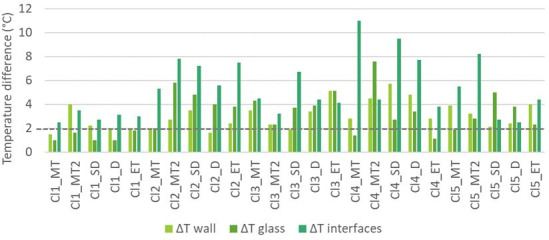
Recorded temperature differences for walls, glass and interfaces during thermal imaging campaign. The horizontal dashed grey line indicates the threshold between higher and lower needs for retrofit, where Δ*T* wall >2°C, there is a higher need for retrofit. For all clusters, the following nomenclature is used: Cln_MT: cluster *n* mid-terrace; Cln_MT2: cluster *n* mid-terrace 2; Cln_SD: cluster *n* semi-detached; Cln_D: cluster 1 detached; Cln_ET: cluster *n* end-terrace, where *n* is the cluster number.

A difference of temperature (Δ*T*) was extracted for the solid wall, the glass and the window and wall interfaces (figure 6). Based on the fact that intervention to the walls would potentially be the most challenging, the solid wall temperature was used as an indicator to define priorities for retrofit. Specifically, where Δ*T* wall >2°C, there is a higher need for retrofit. This indicator appeared to be larger in clusters 3, 4 and 5. Overall, 18 typologies in the whole sample have a higher need for retrofit.

#### 3.1.4. Identifying hard-to-decarbonize dwellings

**Figure 7.**
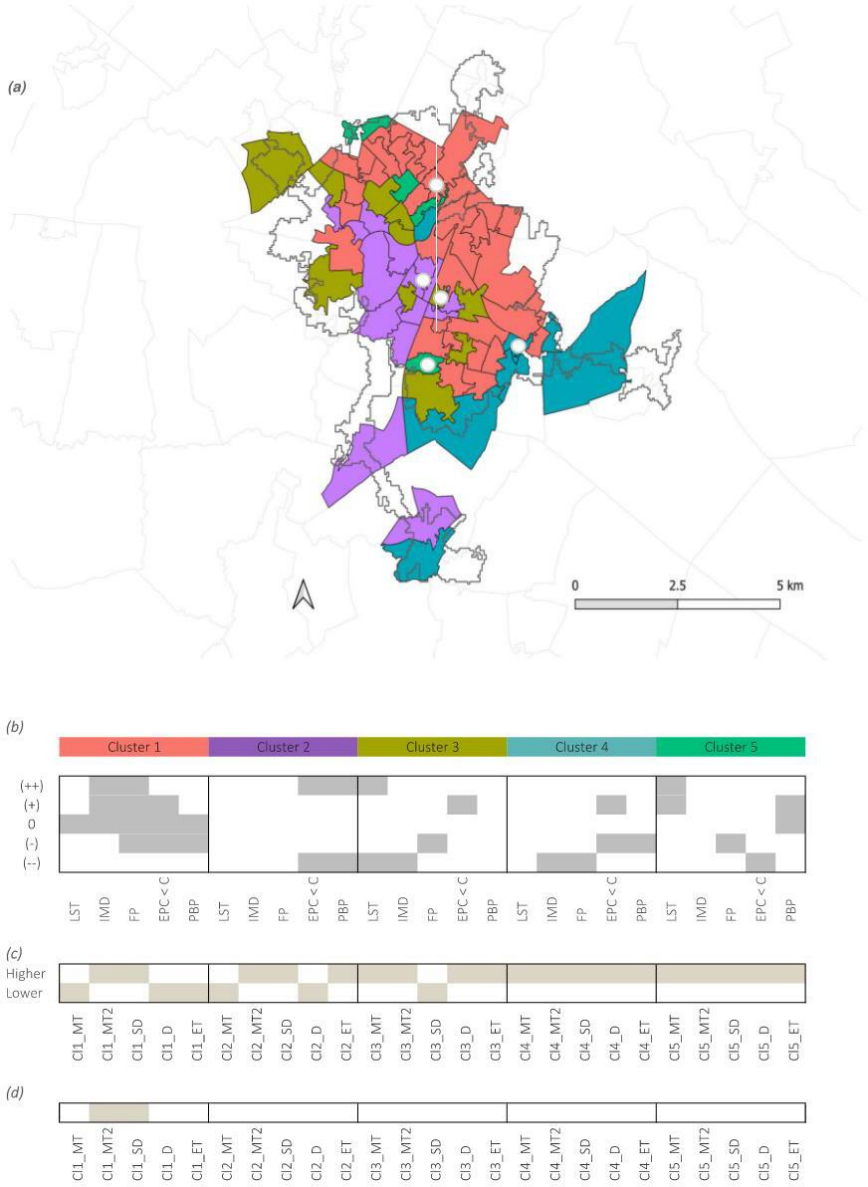
Matrix of IR thermal images collected for five different building for each cluster. LST: land surface temperature; IMD: index of multiple deprivation; FP: percentage of households that are fuel poor; EPC < C: percentage of homes with EPC rating below C; PBP: predominant building period. (*a*) Cambridge map showing the five clusters. White dots indicate areas where thermal imaging was collected for each cluster. (*b*) Clustering enrichment matrix. Grey shaded areas indicate variable categories that are over-represented in each cluster compared to all other clusters. (++) = very high, (+) = high, 0 = medium, (−) = low, (−−) = very low, where very high indicates highest need for attention. (*c*) Building typology retrofit priority. Shaded areas indicate a higher retrofit priority category while unshaded areas suggest lower retrofit need. (*d*) Hard to decarbonize classification. Shaded areas indicate that a building type is hard to decarbonize. For all clusters, the following nomenclature is used: Cln_MT: cluster *n* mid-terrace; Cln_MT2: cluster *n* mid-terrace 2; Cln_SD: cluster *n* semi-detached; Cln_D: cluster 1 detached; Cln_ET: cluster *n* end-terrace, where *n* is the cluster number.

Based on the clustering enrichment at the neighbourhood scale and on the retrofit priorities on a building typology basis, an approach to identify HtD was defined. In detail, typologies located in clusters with an over-representation of IMD and FP between medium to very high and with a higher need for retrofit were defined as HtD (figure 7). Overall, Cl1 MT2 and Cl1 SD are HtD.

#### 3.1.5. Temperature variability and attribute trends

**Figure 8.**
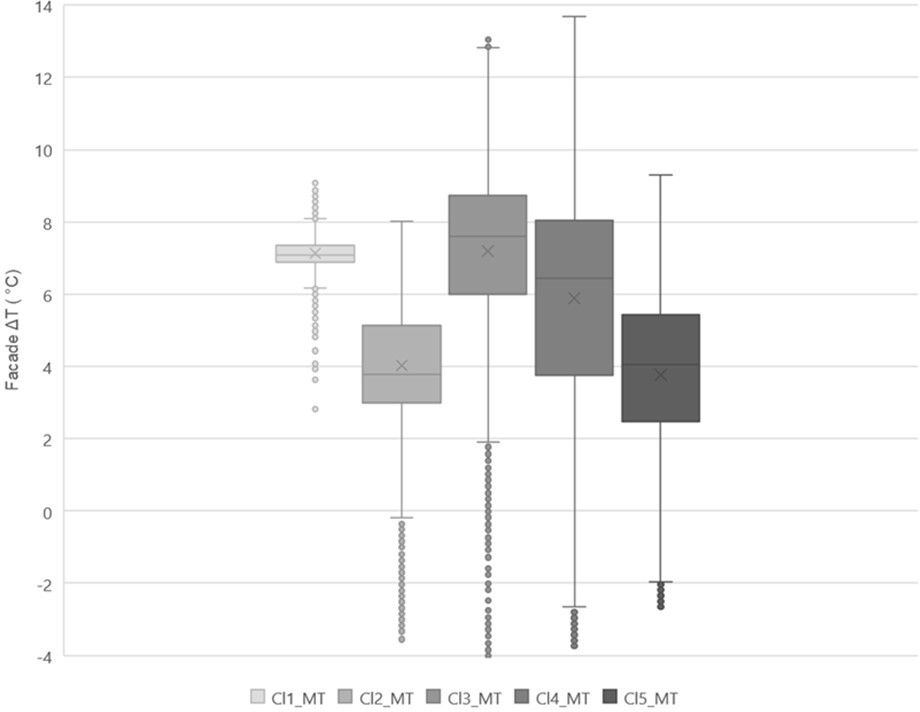
Facade Δ*T* for mid-terrace typology in the different clusters Cln_MT: cluster *n* mid-terrace where *n* is the cluster number.

To understand how the residential units perform in different neighbourhoods, the facade Δ*T* for mid-terrace housing in different clusters was assessed (figure 8). The largest thermal variability was observed for Cl4_MT, followed by Cl5_MT and Cl3_MT. In contrast, the lowest Δ*T* is for Cl1_MT and Cl2_MT. Overall, Cl1_MT facade Δ*T* was the lowest, just under 2°C, and the Cl4_MT facade Δ*T* was the highest, above 14°C.

**Figure 9.**
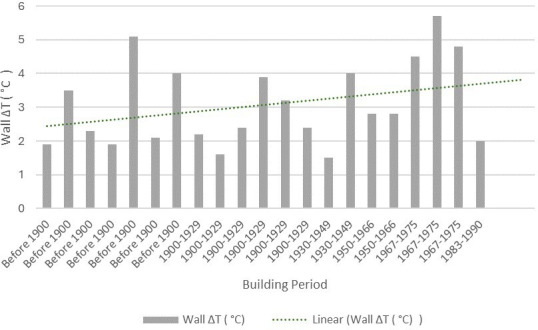
Wall Δ*T* by building period. The graph only includes wall Δ*T* for dwellings for which age was available on the ‘Energy Performance of Building Data: England and Wales’ site by the Department for Levelling Up.

To understand the relationship between the building age and the collected building data, the wall temperature difference and building period were analysed based on public data in the EPC database (figure 9). Most of the wall Δ*T* range from about 2°C to 4°C . However, a higher temperature variability of about 4.5°C is identified for a dwelling dated before 1900. Similarly, a wall Δ*T* of 5.7°C and 4.8°C is observed for two houses from 1967 to 1975, respectively.

**Figure 10.**
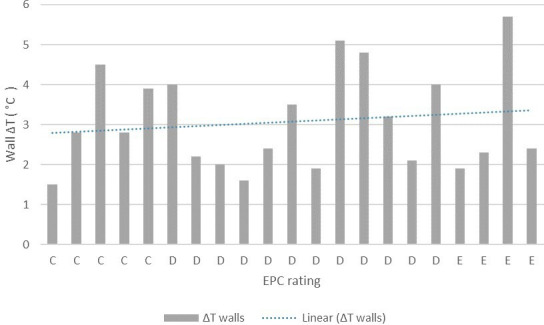
Wall and interface Δ*T* by EPC rating. The graph only includes wall Δ*T* for dwellings for which an EPC rating was available in the ‘Energy Performance of Building Data: England and Wales’ site by the Department for Levelling Up.

Similarly, the relationship between EPC ratings and wall temperature variability was analysed (figure 10). Of note, for the units studied with publicly available EPC rating, C, D and E are the main categories where D is predominant. Wall Δ*T* ranges from 1.5°C to 4.8°C, from 1.9°C to 8.2°C and from 3.6°C to 9.8°C for EPC ratings C, D and E, respectively.

## 4. Discussion

Targeting specific urban areas and dwellings for the implementation of retrofit plans remains one of the most significant challenges for the decarbonization of the built environment. To this end, this work develops and tests a framework methodology to answer *what housing building stock should be targeted for mass retrofit*. Based on a holistic multi-variate and multi-scale approach, it informs the identification of mass retrofit priorities of neighbourhood clusters, of cluster-specific building types and, ultimately, of HtD typologies.

While Cambridge is used as a case study, the approach tested has the potential to be implemented in any other city in the UK and beyond. This research proposes a sequenced methodology to target neighbourhoods and buildings for retrofit at scale within a specific urban context. It is based on a holistic range of variables that inform aspects of the environmental, social and economic performance of the BS and its population. In addition, the recommended framework integrates data at both the neighbourhood and the building scale. Of relevance, it has potential to be extended based on alternative combinations of variables or on other cities in and outside of the UK.

### 4.1. Characteristics of five group areas in Cambridge

At the neighbourhood scale, MCA, AHC and CE allowed the characterization of different Cambridge BS clusters based on a holistic set of variables used. Results suggest that cluster 1, mostly concentrated in the north and east of the city, requires the most attention and financial support from local authorities, showing the highest levels of deprivation and incidence of fuel poor homes. Its building fabric appears to be mostly dated between 1900 and 1982, suggesting potentially different challenges in terms of fabric retrofit measures.

Cluster 2 corresponds to group of neighbourhoods at the centre and west of Cambridge. It is defined by contrasting groups in terms of building predominant period and energy efficiency (EPC < C). In this case, to target mass retrofit strategies, there is potential to interrogate the extremes in this cluster BS which suggests at least two sub-groups: one with recent buildings with adequate energy performance rating and one with significantly older and poor performing buildings. As a result, a fabric first and making visible approaches may be appropriate urban leadership strategies to retrofit these areas, respectively.

Cluster 3 is scattered throughout Cambridge compared with other clusters that are geographically compact. Results suggest that it includes areas with high and low surface temperatures in the context of Cambridge while deprivation and fuel poverty levels are in the lower range. Of note, the energy efficiency requires attention. As such, contrasting conditions can be investigated further from a building fabric and an urban form perspective to help tailor retrofit measures. Potentially, a combination of building envelope and technology interventions is the best suited strategy for this cluster.

Cluster 4 is mostly localized at the city’s southeast edges and is characterized by different levels of EPC rating with the BS being relatively new. Similar to cluster 3, results indicate that it might require less economic support from local authorities based on fuel poverty and deprivation indicators which are among the lowest in the city, in contrast to cluster 1. In this case, the possibility for residents to fund their house retrofit could be explored further. To achieve this, retrofit-related awareness needs to be raised among residents of the area by making the new BS visible to the local government and the general population (e.g. community engagement events, government incentives to retrofit, etc.).

Cluster 5 is characterized by having the lowest geographical extension; only four LSOAs belong to this group. Surface temperatures and building ages in this sub-division are in the high end, suggesting thermal stress. Therefore, there is potential for local authorities to explore the deployment of new system technologies in parallel with a fabric focused approach. Yet, there is an indication of appropriate energy efficiency and lower levels of fuel poverty which could also mean less need for subsidy from the city councils. Similar to cluster 4, a partially resident-funded retrofit can be considered.

Overall, the approach defined and tested here demonstrates its potential to identify distinct urban areas with varied needs for retrofit intervention. The outputs of the analysis can be of significant value for informed decision-making by local authorities. However, the limitations of the clustering method should be considered.

### 4.2. Limitations of the clustering approach

Ideally, the area extension of the multiple clusters identified should be similar to help local authorities target urban intervention. Yet, in the context of this work, urban groups are varied in size. Indeed, a single cluster can relate to a significant geographic area (e.g. cluster 1) or can be dispersed throughout the city (e.g. clusters 3 and 4). The presented methodology could be explored further with a focus on subgrouping strategies and adjusted clustering. This could be done, for instance, by developing sensitivity analysis based on community consultation and further understanding of any LSOA outliers. In this instance, a staged clustering could be conducted providing higher granularity of the city’s need for mass retrofit.

On the other hand, complementing the analysis with additional variables of relevance can improve the robustness of the method. For instance, more specific energy and thermal performance, fabric characteristics at scale, among others, could be considered. Nonetheless, this is limited by either the existence or, in some cases, the public availability of the data. Greater efforts to produce and disclose such data would contribute to effectively target retrofits at scale.

### 4.3. Building scale analysis

Most of the typologies in the sample analysed through thermal imaging appear to be in need of urgent retrofit based on the campaign conducted. Of note, every house surveyed in clusters 4 and 5 and some others in other groups are in need of imminent retrofit. In detail, the large majority of the sample results indicate wall temperature difference above 2°C, indicating heat loss. In addition, the IR data show consistent anomalies such as thermal bridges, insulation missing or air leakage throughout the sample. Overall, for building types representative of single-family housing in Cambridge, construction anomalies and the prevalent urgency to retrofit are characteristic of the sample studied.

The data also suggest that the wall temperature difference—here used as an indicator of the need for retrofit—is correlated with the EPC rating of the residential units analysed. Specifically, when the EPC rating of the residential unit is worse, the wall Δ*T* tends to be higher based on a linear trend. However, the building sample analysed only covered EPC ratings ranging from C to E, but no buildings with EPCs A, B or F were surveyed. The correlation between EPCs and temperature difference can be explored further based on a more comprehensive sample that includes all EPC ratings.

Unexpectedly, a positive correlation between the building age and the wall Δ*T* is not identified. Older construction methods would typically include solid walls while newer building fabric tends to feature cavity walls. Heat loss tends to be quicker and higher in the first than in the second. The lack of correlation can be explained to some extent by the fact that the large majority of the sample studied features solid wall construction and is dated prior to the 1950s. In this case, the fabric build up temperature difference may be reduced faster than recent constructions that include more facade layers. To assess detailed retrofit interventions with the use of thermal imaging, it is relevant to consider a larger variety of building ages and wall types.

### 4.4. Limitations of the building analysis

While the selection of buildings to undertake the thermal imaging campaign followed specific criteria, it may not be representative of the diversity of the different urban areas and building stock in Cambridge. Similar orientation, footprint, availability of an EPC and the location in the neighbourhood groups were criteria considered to identify buildings where possible. However, the selection of houses may be a limited representation of the entirety of each cluster. In fact, despite being in different clusters, there was some proximity between the neighbourhoods where the houses were located, for instance clusters 1 and 2. This could be addressed by using IR methods with a larger scope like the use of cars in thermal campaigns. Of relevance, a broader range of residential typologies could be studied based on the specific urban context. Importantly, building physics analysis could be implemented to inform the wall temperature difference considered for the definition of retrofit priorities.

Furthermore, the possibility for the IR building scale to inform neighbourhood scale clusters is limited due to the number of dwellings surveyed per group. To define retrofit priorities among building typologies in the different clusters, IR building unit data were collected and analysed with a focus on five buildings per cluster. This approach can be expanded through the use of tools that allow the upscaling of data collection including thermal imaging cameras carried by drones or cars. Similarly, desktop thermal modelling can support the understanding of dwellings’ thermal performance. Importantly, such methods could enable a more comprehensive characterization of full neighbourhoods’ housing to target mass retrofit strategies accurately.

In addition, the operation of the building system, including occupancy schedule and thermostat settings, was unknown during the data collection and could not be accounted for in the data analysis. Ideally, the IR campaign should be undertaken on houses with similar conditions. However, this is not always possible and could be addressed through surveys that inform general heating usage patterns.

### 4.5. Hard to decarbonize

This work proposes a method to identify HtD homes in Cambridge by bridging both the neighbourhood- and the building-scale data. Specifically, a building typology with high need for retrofit is considered to be HtD when it belongs to a neighbourhood where IMD and FP are of concern. In detail, mid-terraced and semi-detached typologies in cluster 1 are found to be HtD. Here, focused financial support from local authorities for retrofitting appears to be most needed to tackle the broader retrofit challenge.

The approach to identifying HtD in this work overlaps to some extent with initial definitions in the literature. Indeed, while construction and age data are available for only three of the five typologies, characteristics appear to be consistent. Specifically, these houses were constructed prior to the 1950s and have a solid wall construction with no insulation, falling into the HtT category, as defined by Ambrose & Raslan [8], of those that are difficult to decarbonize. Here, part of the building upgrade challenge lies on the wall construction and the limited space and potential to insulate. To this end, building fabric focused interventions that consider low thickness thermal insulation could be explored further.

## 5. Conclusion

Mass retrofit or retrofit at scale is an area of continued growth in the UK and beyond. It refers to the upgrade of multiple buildings at the same time, rather than on a unit-by-unit basis. The need for an upscale is partially based on time and quantum pressure to achieve carbon emission reduction on the residential building stock. Multiple literature sources converge in that the neighbourhood scale is the focus for decarbonization [42]. Of relevance, this research focuses on identifying the existing residential building stock that should be targeted for retrofit at scale.

The contribution of our work is twofold. First, a holistic multi-variate and multi-scale approach informs the identification of retrofit at scale priorities which is relevant for the academic field. Second, the interpretation of results has the potential to inform urban leadership decision-making and retrofit policy. Overall, through a holistic and integrated analysis, the work demonstrates a procedure to characterize the existing building stock and inform intervention strategies.

Our work effectively develops and tests a methodology to target retrofit at scale; Cambridge is used as a case study. First, the framework enables a holistic approach to building upgrade at scale integrating environmental, social and economic aspects of the existing built fabric and of its population. Second, the resulting identification and grouping of neighbourhoods informs retrofit priorities which are relevant for built environment practitioners and, especially, for local authorities. Third, the sequenced method approach is comprehensive and considers data at both the neighbourhood and building scale. Fourth, such integration of scale analysis resulted in the definition of what building stock is HtD which is of relevance for local authorities to target policy strategies. Overall, the approach tested allows one to respond to the research question *what housing building stock should be targeted for mass retrofit*.

In the context of Cambridge, one of five groups of neighbourhoods is identified to require more financial support from government for the implementation of retrofit strategies. It is characterized, among others, by higher levels of deprivation and fuel poverty. In addition, single-family housing typologies are categorized based on non-intrusive thermal analysis that informs the need for upgrades. As a result, housing types including mid-terrace, semi-detached, detached and end-terrace are found to be in need of urgent retrofit in different urban areas. All in all, it is only when both neighbourhood and building scale findings are overlayed that the portion of the building stock that is HtD is defined. Here, mid-terrace and semi-detached houses in the economic subsidy priority neighbourhood groups are found to be the most difficult to upgrade.

This framework contributes to the definition of HtD homes. Recent literature attempts to frame what is HtD based on dwelling physical properties and attributes including wall construction type, connection to networks, EPC ratings, levels of fuel poverty, heritage value, among others. Building on this, the methodology here presented combines data analysis at the neighbourhood and building scale that informs need for attention from local authorities and urgency of retrofit, respectively. Yet, future research is required to inform HtD potential interventions and expected performance improvement potential. Being a significant contributor to emissions, HtD should be at the core of wider retrofit urban strategies.

The methodology implemented in this research offers the opportunity for further research with a diverse and adjustable combination of variables that can be adapted to a wide variety of urban decarbonization challenges. The literature shows a consistent use of variables in the study of retrofit that include building age, EPC ratings and typologies, among others. In addition, multi-variate approaches have been implemented in the urban context for different purposes, including the study of the compact city, as an example. Here, with a focus on retrofit at scale, LST, IMD, the percentage of households in fuel poverty, EPC ratings and the predominant period are considered. Expanding these set of inputs, including occupant data, heritage value or metered performance data for example, provides the basis for future investigation. As a result, based on different combination of parameters, different neighbourhood groupings may arise for a given city.

To conclude, building stock mass retrofit can be targeted by analysing and visualizing holistic multi-scale and multi-layered data. While abundant information is already available, joint efforts to increase the expansion of publicly accessible built environment platforms is needed to tackle the urgency of retrofit at scale. As a result, more robust and accurate existing built environment sustainable development strategies can be expected.

## Data Availability

Open data accessed from sources listed in table 2 are used in this study. They can be accessed [43]. Supplementary material is available online [44].

## References

[1] Buildings Editorial Office. 2015 Acknowledgement to reviewers of Buildings in 2014. Buildings **5**, 14–15. (10.3390/buildings5010014)

[2] UKGBC. 2021 Net zero whole life carbon roadmap for the built environment. See https://www.ukgbc.org/ukgbc-work/net-zero-whole-life-roadmap-for-the-built-environment/.

[3] Committee on Climate Change. 2019 UK housing: fit for the future?. See https://www.theccc.org.uk/wp-content/uploads/2019/02/UK-housing-Fit-for-the-future-CCC-2019.pdf.

[4] Wade F, Visscher H. 2021 Retrofit at scale: accelerating capabilities for domestic building stocks. Build. Cities **2**, 800–811. (10.5334/bc.158)

[5] ONS. 2022 Age of the property is the biggest single factor in energy efficiency of homes. See https://www.ons.gov.uk/peoplepopulationandcommunity/housing/articles/ageofthepropertyisthebiggestsinglefactorinenergyefficiencyofhomes/2021-11-01.

[6] Foster S, Tahir F, Orchard K, Walker I, Raslan R, Schwartz Y. 2019 Analysis on abating direct emissions from ‘hard-to-decarbonise’ homes. London, UK: Committee on Climate Change.

[7] Gupta R, Gregg M. 2020 Domestic energy mapping to enable area-based whole house retrofits. Energy Build. **229**, 110514. (10.1016/j.enbuild.2020.110514)

[8] Raslan R, Ambrose A. 2022 Solving the difficult problem of hard to decarbonize homes. Nat. Energy **7**, 675–677. (10.1038/s41560-022-01075-w)

[9] UKGBC. 2022 Climate change: UKGBC’s vision for a sustainable built environment is one that mitigates and adapts to climate change. See https://www.ukgbc.org/climate-change-2/.

[10] National Housing Federation and Local Government Association. 2022 Hard to decarbonise social homes. See https://www.housing.org.uk/globalassets/files/climate-and-sustainability--energy-crisis/hard-to-decarbonise-homes-2022.pdf.

[11] Houghton E, Kelly L, Raslan R, Cui C. 2023 Defining and identifying complex-to-decarbonise homes and retrofit solutions. Research report. See https://assets.publishing.service.gov.uk/media/65819bc3fc07f3000d8d44a5/complex-to-decarbonise-homes-report.pdf.

[12] Hofman P, Wade F, Webb J, Groenleer M. 2021 Retrofitting at scale: comparing transition experiments in Scotland and the Netherlands. Build. Cities **2**, 637. (10.5334/bc.98)

[13] Rosenow J, Kern F, Rogge K. 2017 The need for comprehensive and well targeted instrument mixes to stimulate energy transitions: the case of energy efficiency policy. Energy Res. Soc. Sci. **33**, 95–104. (10.1016/j.erss.2017.09.013)

[14] Brocklehurst F, Morgan E, Greer K, Wade J, Killip G. 2021 Domestic retrofit supply chain initiatives and business innovations: an international review. Build. Cities **2**, 533. (10.5334/bc.95)

[15] Deb C, Schlueter A. 2021 Review of data-driven energy modelling techniques for building retrofit. Renew. Sustain. Energy Rev. **144**, 110990. (10.1016/j.rser.2021.110990)

[16] International Energy Agency. 2018 World Energy Outlook 2018. Paris, France: IEA. (10.1787/weo-2018-en)

[17] Wise F, Moncaster A, Jones D. 2021 Rethinking retrofit of residential heritage buildings. Build. Cities **2**, 495. (10.5334/bc.94)

[18] Gupta R, Gregg M. 2018 Targeting and modelling urban energy retrofits using a city-scale energy mapping approach. J. Clean. Prod. **174**, 401–412. (10.1016/j.jclepro.2017.10.262)

[19] Gregório V, Seixas J. 2017 Energy savings potential in urban rehabilitation: a spatial-based methodology applied to historic centres. Energy Build. **152**, 11–23. (10.1016/j.enbuild.2017.06.024)

[20] Meijer F, Itard L, Sunikka-Blank M. 2009 Comparing European residential building stocks: performance, renovation and policy opportunities. Build. Res. Inf. **37**, 533–551. (10.1080/09613210903189376)

[21] Ben H, Steemers K. 2014 Energy retrofit and occupant behaviour in protected housing: a case study of the Brunswick Centre in London. Energy Build. **80**, 120–130. (10.1016/j.enbuild.2014.05.019)

[22] England H. 2021 Acknowledgment to reviewers of Heritage in 2020. Heritage **4**, 278–280. (10.3390/heritage4010017)

[23] English Heritage. 2011 Energy efficiency and historic buildings: application of part L of the building regulations to historic and traditionally constructed buildings. See http://www.english-heritage.org.uk/partL.

[24] Schwarz PM, Taylor TN. 1995 Cold hands, warm hearth? Climate, net takeback, and household comfort. Energy J. **16**, 41–54. (10.5547/issn0195-6574-ej-vol16-no1-3)

[25] Sun M, Bardhan R. 2024 Identifying hard-to-decarbonize houses from multi-source data in Cambridge, UK. Sustain. Cities Soc. **100**, 105015. (10.1016/j.scs.2023.105015)

[26] Lemonsu A, Viguié V, Daniel M, Masson V. 2015 Vulnerability to heat waves: impact of urban expansion scenarios on urban heat island and heat stress in Paris (France). Urban Clim. **14**, 586–605. (10.1016/j.uclim.2015.10.007)

[27] Templeton G, Taleghani M. 2024 Analysis of micro and macro urban heat islands in an industrial city: Bradford, UK. Nat. Based Sol. **5**, 100124. (10.1016/j.nbsj.2024.100124)

[28] Bardhan R, Kurisu K, Hanaki K. 2015 Does compact urban forms relate to good quality of life in high density cities of India? Case of Kolkata. Cities **48**, 55–65. (10.1016/j.cities.2015.06.005)

[29] Cambridgeshire County Council. 2016 Cambridge city housing stock condition atlas and ranking tool. See https://cambridgeshireinsight.org.uk/wp-content/uploads/2018/02/StockConditionRanking-tool-background.pdf.

[30] Ministry of Housing, Communities and Local Government. 2019 The English Indices of Deprivation 2019 (IoD2019). See https://assets.publishing.service.gov.uk/media/5d8e26f6ed915d5570c6cc55/IoD2019_Statistical_Release.pdf.

[31] Yu Y, Liu Y, Yu P, Liu Y, Yu P. 2018 Land surface temperature product development for JPSS and GOES-R missions. In Comprehensive remote sensing, pp. 284–303. Amsterdam, The Netherlands: Elsevier. (10.1016/b978-0-12-409548-9.10522-6)

[32] Ermida SL, Soares P, Mantas V, Göttsche FM, Trigo IF. 2020 Google Earth Engine open-source code for land surface temperature estimation from the Landsat series. Remote Sens. **12**, 1471. (10.3390/rs12091471)

[33] Department for Business, Energy & Industrial Strategy. 2022 Sub-regional fuel poverty in England (2020 data). See https://www.gov.uk/government/statistics/sub-regional-fuel-poverty-data-2022.

[34] DCLG. 2017 A guide to energy performance certificates for the marketing, sale and let of dwellings: improving the energy efficiency of our buildings. London, UK: Department for Communities and Local Government.

[35] Costa PS, Santos NC, Cunha P, Cotter J, Sousa N. 2013 The use of multiple correspondence analysis to explore associations between categories of qualitative variables in healthy ageing. J. Aging Res. **2013**, 302163. (10.1155/2013/302163) 24222852 PMC3810057

[36] Hong Y, Ezeh CI, Zhao H, Deng W, Hong SH, Tang Y. 2021 A target-driven decision-making multi-layered approach for optimal building retrofits via agglomerative hierarchical clustering: a case study in China. Build. Environ. **197**, 107849. (10.1016/j.buildenv.2021.107849)

[37] Subramanian A *et al*. 2005 Gene set enrichment analysis: a knowledge-based approach for interpreting genome-wide expression profiles. Proc. Natl Acad. Sci. USA **102**, 15545–15550. (10.1073/pnas.0506580102) 16199517 PMC1239896

[38] Climate Editorial Office. 2015 Acknowledgement to reviewers of Climate in 2014. Climate **3**, 133–134. (10.3390/cli3010133)

[39] Low Energy Transformation Initiative. 2021 Climate emergency retrofit guide: how existing homescan be adapted to meet UK climate targets. See https://www.leti.uk/_files/ugd/252d09_c71428bafc3d42fbac34f9ad0cd6262b.pdf.

[40] Sadhukhan D *et al*. 2020 Estimating surface temperature from thermal imagery of buildings for accurate thermal transmittance (U-value): a machine learning perspective. J. Build. Eng. **32**, 101637. (10.1016/j.jobe.2020.101637)

[41] Resende MM, Gambare EB, Silva LA, Cordeiro YdS, Almeida E, Salvador RP. 2022 Infrared thermal imaging to inspect pathologies on façades of historical buildings: a case study on the Municipal Market of São Paulo, Brazil. Case Stud. Constr. Mater. **16**, e01122. (10.1016/j.cscm.2022.e01122)

[42] Reinhart C, Dogan T, Jakubiec A, Rakha T, Sang A. 2013 Umi—an urban simulation environment for building energy use, daylighting and walkability. In 13th Conf. of International Building Performance Simulation Association, Chambéry, France, 26–28 August 2013, pp. 476–483. (10.26868/25222708.2013.1404)

[43] Mora H, Bardhan R. 2024 Towards carbon neutrality: mapping mass retrofit opportunities in Cambridge, UK. Dryad Digital Repository. [Dataset] (10.5061/dryad.3tx95x6r0) PMC11732419 39816744

[44] Mora H, Bardhan R. 2025 Supplementary material from: Towards Carbon Neutrality: Mapping Mass Retrofit Opportunities in Cambridge, UK. FigShare (10.6084/m9.figshare.c.7618561) PMC11732419 39816744

